# Bacterial endophthalmitis caused by an intraocular cilium in a patient under treatment with infliximab

**DOI:** 10.1186/1869-5760-3-50

**Published:** 2013-06-05

**Authors:** Xue-hai Jin, Kenichi Namba, Wataru Saito, Daiju Iwata, Susumu Ishida

**Affiliations:** 1 Department of Ophthalmology, Hokkaido University Graduate School of Medicine, Sapporo, Japan

**Keywords:** Cilium, Endopthalmitis, Anti-tumour necrosis factor-α antibody therapy, Crohn’s disease

## Abstract

**Background:**

We report a case of bacterial endophthalmitis caused by an intraocular cilium in a patient without any history of trauma or ocular surgery.

**Findings:**

A 32-year-old Caucasian male showed symptoms of orbital myositis and scleritis, with no intraocular inflammation in the right eye. The patient had been treated with infliximab for Crohn’s disease. Three weeks after initiation of oral prednisolone therapy, he developed bacterial endophthalmitis. During pars plana vitrectomy, a cilium in the massive vitreous opacity was found. A focal scleral necrosis was detected just outside where the cilium was intraoperatively observed. Vitreous culture showed the presence of *Staphylococcus aureus*.

**Conclusions:**

The intraocular cilium seemed to be the aetiology of the endophthalmitis in this case, which suggests that anti-tumour necrosis factor-α therapy may play a role in the migration of cilia into the globe and the occurrence of endophthalmitis.

## Findings

### Introduction

The presence of intraocular cilia is rare but is sometimes reported as a complication from a penetrating ocular injury. The response of the eye to the retained intraocular cilia may be an early severe inflammation or delayed inflammatory reaction in the form of plastic iridocyclitis, granulomatous inflammation or foreign-body reaction [1]. Up to now, there are only three case reports in the literature of an intraocular cilium without any history of trauma or surgery; there was no evidence of an external entrance site for the intraocular cilium detected in these three cases [2-4].

We present a rare case of bacterial endophthalmitis suspected to be caused by an intraocular cilium without any history of penetrating ocular trauma or ocular surgery. The cilium is speculated to migrate gradually into the vitreous cavity via necrotised sclera induced by chronic infection, finally to cause exogenous endophthalmitis. This case was treated with anti-tumour necrosis factor (TNF)-α antibody for Crohn’s disease before the onset of endophthalmitis.

## Case report

A 32-year-old Caucasian male complained of redness with tenderness that lasted for 3 days in his right eye. He received oral prednisolone therapy for 2 weeks on the diagnosis of presumed orbital myositis without intraocular abnormality in an eye clinic. However, his symptoms gradually worsened, so he was referred to our hospital. He had been treated with infliximab, an anti-TNF-α antibody, for 2 years for Crohn’s disease, and the disease was well controlled. There was no history of trauma or ocular surgery.

**Figure 1:**
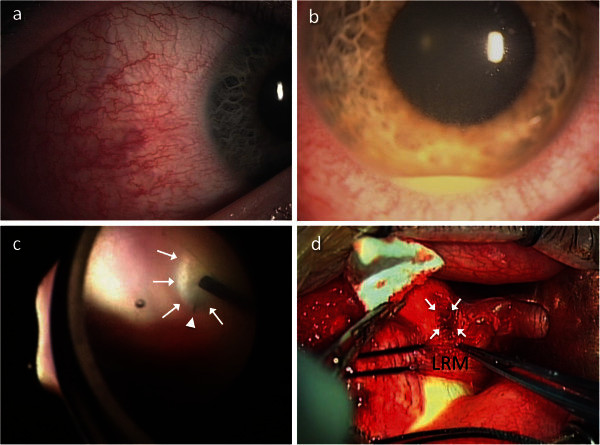
**Photographs of the right eye.** Photographs of the right eye showing marked temporal conjunctival and scleral injections at the first visit (**a**) and anterior chamber inflammation with hypopyon 1 week after the first visit (**b**). Intraoperatively, a yellow-white massive vitreous opacity (white arrows) was observed at the supero-temporal retina and a foreign body (white triangle) resembling a cilium was found in the vitreous opacity (**c**). A focal scleral necrosis (arrows) was detected just outside where the foreign body was observed (**d**). LRM, lateral rectus muscle.

His decimal visual acuity was 1.0 OU. Slit-lamp examination revealed marked temporal conjunctival and scleral injections OD, with no anterior chamber inflammation (Figure 1a). Funduscopic examination revealed a tiny creamy intraretinal infiltration at the supero-temporal periphery without vitreous opacity OD. Nothing appeared abnormal at OS. Ultrasonic B-mode imaging and MRI showed no abnormalities. The white blood cell count and C-reactive protein level were within normal limits.

**Figure 2:**
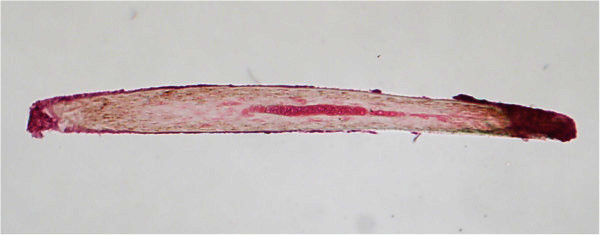
Histopathological examination confirming the foreign body as a human hair (hematoxylin and eosin, ×4).

Administration of prednisolone (40 mg/day) was orally initiated for scleritis. However, he returned 1 week later with complaints of blurred vision and exacerbated pain OD. Examination revealed a visual acuity of 1.0, 3+ cells in the anterior chamber and massive vitreous opacity above the temporal intraretinal infiltrate OD. On the following day, his visual acuity had deteriorated to hand motion OD. Anterior chamber inflammation worsened to 4+ cells with hypopyon (Figure 1b), and the fundus was invisible due to exacerbated vitreous opacity. As suspicious endophthalmitis was diagnosed, he immediately underwent pars plana vitrectomy. Intraoperatively, a yellow-white mass, suggestive of subretinal abscess, was observed at the retina, peripheral to the pars plana of the supero-temporal side; and a foreign body resembling a cilium was found in the massive vitreous opacity (Figure 1c). A focal scleral necrosis was detected at exactly the same site where the foreign body was observed (Figure 1d). Histopathological examination later confirmed the foreign body to be a human hair (cilium) (Figure 2). Vitreous culture showed the presence of *Staphylococcus aureus*. After the surgery, endophthalmitis became silent with the treatment of intravenous antibiotics; however, the retina was partially detached due to the occurrence of secondary proliferative vitreoretinopathy.

## Discussion

We encountered a case of bacterial endophthalmitis caused by an intraocular cilium. This case was unique because of the absence of any history of penetrating ocular trauma and the history of treatment with an anti-TNF-α antibody for Crohn’s disease. Although the possibility of remotely unrecalled ocular trauma may have been involved in the cilium migration, there were no intraocular foreign bodies except the cilium detected during surgery. Moreover, the clinical course of scleritis - without intraocular inflammation antecedent to the onset of endophthalmitis - suggests that it may take time for the cilium migration, rather than a sudden trauma. During surgery, a focal scleral necrosis was found at the same site where the cilium was detected, suggesting that necrotized sclera gave the migrating cilium entrance to the globe. Such conditions are unlikely to be due to a sudden penetrating ocular trauma. We speculate that the accidentally migrated cilium under the conjunctiva caused chronic bacterial infection and then focal scleral necrosis and that subsequently, the cilium migrated into the vitreous cavity via the necrotised sclera, finally to cause exogenous endophthalmitis.

Cilium migration into the subconjunctival space usually does not cause chronic infection in immunocompetent people because microorganisms around the cilia are eliminated easily with innate immune responses. In this case, the history of treatment with infliximab, an anti-TNF-α antibody, may play a role in the migration of the intraocular cilium. TNF-α is a central key cytokine that mediates inflammatory response. Therefore, the anti-TNF-α drugs are highly effective treatment for chronic inflammatory diseases such as rheumatoid arthritis and Crohn’s disease. Meanwhile, TNF-α is also an important cytokine that plays a central role in the innate immune response towards microorganisms [5]. The immune response eliminates them by inducing strong inflammation. However, under treatment with an anti-TNF-α drug, microorganisms survive easily, and the focal infection tends to persist with a mild inflammation [6]. In this case, therefore, we speculate that a chronic infection in the subconjunctival space is prolonged due to the increased vulnerability to infection by the anti-TNF-α drug administration, which led to focal scleral necrosis subsequently.

This case indicates that intraocular cilia may be an aetiology of endophthalmitis even in patients without any history of trauma or surgery. Furthermore, anti-TNF-α drug therapy may play a role in the migration of the intraocular cilium and the development of endophthalmitis.

## Abbreviations

TNF: Tumour necrosis factor.

## Competing interests

All authors declare that they have no competing interest.

## Authors’ contributions

XJ managed the case and drafted the manuscript. KN managed the case, also participated in the design of the study and revised the manuscript critically. WS was involved in treating the case, also reviewed the paper and gave valuable comments. DI was involved in the medical care of the case and contributed to the collection of data. SI supervised the management of the case, also revised the manuscript and made the best possible comments. All authors read and approved the final manuscript.
